# The fibrinogen-to-albumin ratio is associated with intracranial atherosclerosis plaque enhancement on contrast-enhanced high-resolution magnetic resonance imaging

**DOI:** 10.3389/fneur.2023.1153171

**Published:** 2023-05-24

**Authors:** Ye Li, Yuxuan Feng, Rui Liu, Meijuan Dang, Tao Li, Lili Zhao, Jialiang Lu, Ziwei Lu, Yang Yang, Xiaoya Wang, Yating Jian, Heying Wang, Wei Huang, Lei Zhang, Guilian Zhang

**Affiliations:** ^1^Department of Neurology, The Second Affiliated Hospital of Xi'an Jiaotong University, Xi'an, Shaanxi, China; ^2^Department of Neurology, The First Hospital of Yulin, Yulin, Shaanxi, China; ^3^Department of Medical Imaging, The Second Affiliated Hospital of Xi'an Jiaotong University, Xi'an, Shaanxi, China

**Keywords:** intracranial atherosclerotic stenosis, high-resolution magnetic resonance imaging, plaque enhancement, inflammation, fibrinogen-to-albumin ratio

## Abstract

**Background:**

Contrast-enhanced high-resolution magnetic resonance imaging (CE-HR-MRI) is a useful imaging modality to assess vulnerable plaques in intracranial atherosclerotic stenosis (ICAS) patients. We studied the relationship between the fibrinogen-to-albumin ratio (FAR) and plaque enhancement in patients with ICAS.

**Methods:**

We retrospectively enrolled consecutive ICAS patients who had undergone CE-HR-MRI. The degree of plaque enhancement on CE-HR-MRI was evaluated both qualitatively and quantitatively. Enrolled patients were classified into no enhancement, mild enhancement, and obvious enhancement groups. An independent association of the FAR with plaque enhancement was identified by multivariate logistic regression and receiver operating characteristic (ROC) curve analyses.

**Results:**

Of the 69 enrolled patients, 40 (58%) were classified into the no/mild enhancement group, and 29 (42%) into the obvious enhancement group. The obvious enhancement group had a significantly higher FAR than the no/mild enhancement group (7.36 vs. 6.05, *p* = 0.001). After adjusting for potential confounders, the FAR was still significantly independently associated with obvious plaque enhancement in multiple regression analysis (odds ratio: 1.399, 95% confidence interval [CI]: 1.080–1.813; *p* = 0.011). ROC curve analysis revealed that FAR >6.37 predicted obvious plaque enhancement with 75.86% sensitivity and 67.50% specificity (area under the ROC curve = 0.726, 95% CI: 0.606–0.827, *p* < 0.001).

**Conclusion:**

The FAR can serve as an independent predictor of the degree of plaque enhancement on CE-HR-MRI in patients with ICAS. Also, as an inflammatory marker, the FAR has potential as a serological biomarker of intracranial atherosclerotic plaque vulnerability.

## 1. Introduction

Intracranial atherosclerotic stenosis (ICAS) is one of the main causes of ischemic stroke worldwide, especially in Chinese populations; it accounts for about 33–50% of all strokes and more than half of all cases of transient ischemic attack (TIA) (1, 2). Compared with other causes of stroke, in ICAS recurrence of ischemic stroke is more likely (3, 4). Recurrence of ICAS imposes a substantial economic burden on patients and society (5). Inflammation plays a major role in the progression of atherosclerosis and atherosclerotic plaque rupture (6).

In recent years, more emphasis has been placed on contrast-enhanced high-resolution magnetic resonance imaging (CE-HR-MRI), which is a non-invasive imaging modality that can reliably visualize extracranial-intracranial artery wall pathologies and reveal atherosclerotic plaque composition and morphology (7). As such, CE-HR-MRI is useful for assessing plaque vulnerability (8). Previous studies suggested that extracranial artery plaque enhancement after contrast agent injection is associated with inflammation inside plaques and neovascularization, which increases endothelial permeability (9–11). However, few studies have investigated the association between plaque enhancement in ICAS and serum inflammatory markers.

Fibrinogen is not only a key enzyme in the coagulation cascade, but also a potent marker of inflammation. A recent large registry-based study showed that higher plasma fibrinogen levels were associated with worse functional outcomes for ischemic stroke and TIA patients (12). In contrast, serum albumin has anti-inflammatory and antioxidant properties. Low albumins levels were related to poor outcomes in ischemic stroke patients (13). The fibrinogen-to-albumin ratio (FAR), as a simple and inexpensive method for assessing inflammation, is attracting attention as a new prognostic marker for ischemic stroke (14, 15) and may be able to predict the long-term risk of cardiovascular events (16, 17). However, the link between the FAR and plaque enhancement in ICAS patients remains unclear.

The main purpose of this study was to investigate the relationship between the FAR and plaque enhancement among ICAS patients. It is hoped that this study will facilitate the identification of unstable plaques, to better stratify patients and guide therapeutic management.

## 2. Methods

### 2.1. Patients

This retrospective observational study enrolled 112 consecutive patients with ICAS who underwent CE-HR-MRI examinations at the Second Affiliated Hospital of Xi’an Jiaotong University between January 2022 and September 2022.

The inclusion criteria were as follows: 1) underwent an CE-HR-MRI examination, 2) ICAS confirmed by magnetic resonance angiography or digital subtraction angiography, and 3) at least one risk factor for atherosclerosis, such as hypertension, hyperlipidemia, diabetes mellitus, coronary heart disease, obesity, or smoking. The exclusion criteria were as follows: 1) asymptomatic ICAS, 2) culprit plaque located in the extracranial part of the internal carotid artery or vertebral artery, 3) non-atherosclerotic vasculopathy contributing to stroke or TIA, such as atrial fibrillation, artery dissection, moyamoya disease, or vasculitis; 4) infarcts in multiple vascular territories, 5) liver disease, malignant tumors, or autoimmune disease as a comorbidity, and 6) poor-quality images or presence of artifacts. As this was a retrospective study that analyzed anonymized data, the requirement for informed consent was waived by the institutional review board.

### 2.2. Data collection

We retrospectively collected patients’ demographic information, vascular risk factors, laboratory test, and imaging information data from their medical records. Each patient underwent a 12-lead electrocardiogram immediately after admission to the hospital, and Holter monitoring was performed if necessary. Blood samples were collected and processed within 24 h of hospital admission. Plasma fibrinogen was measured using an automatic coagulation analyzer (CS-5100; Sysmex, Kobe Japan). Albumin levels were measured using an automatic biochemical analyzer (AU5800; Beckman Coulter, Pasadena, CA, United States). The FAR was calculated by dividing the fibrinogen level by the serum albumin level. The neutrophil-to-lymphocyte ratio was calculated by dividing the neutrophil count by the lymphocyte count. The lymphocyte-to-monocyte ratio (LMR) was calculated by dividing the lymphocyte count by the monocyte count. Finally, the systemic immune-inflammation index (SII) was calculated as (N × P)/L, where N, P, and L represent the neutrophil count, platelet count and lymphocyte count, respectively.

### 2.3. MRI protocols

MRI was performed using a 3.0 T scanner (Signa Pioneer; GE Healthcare, Milwaukee, WI, United States) with a 21-channel head/neck coil. The scanning sequences included three-dimensional time-of-flight MR angiography (3D TOF-MRA), pre/postcontrast enhanced T1 weighted imaging (T1WI) with 3D fast spin echo and extended echo train acquisition (CUBE), and proton density weighted imaging with CUBE (CUBE PDWI). The scanning parameters were as follows: 1) 3D TOF-MRA: repetition time (TR)/echo time (TE) = 18 ms/2.7 ms, field of view (FOV) = 220 × 220 mm, matrix size = 416 × 224, slice thickness = 1.4 mm, acquisition time = 1′56″, 2) CUBE T1WI: TR/TE = 700 ms/minimum, FOV = 220 × 220 mm, matrix size = 320 × 288, slice thickness = 0.8 mm, acquisition time = 6′30″, and 3) CUBE PDWI: TR/TE = 1,500 ms/12.1 mm, FOV = 220 × 220 mm, matrix size = 320 × 288, slice thickness = 0.8 mm, acquisition time = 4′30″. TOF-MRA was mainly used to identify the location and extent of stenosis. CE CUBE T1WI was performed 5 min after contrast agent injection.

### 2.4. Image analysis

**Figure 1:**
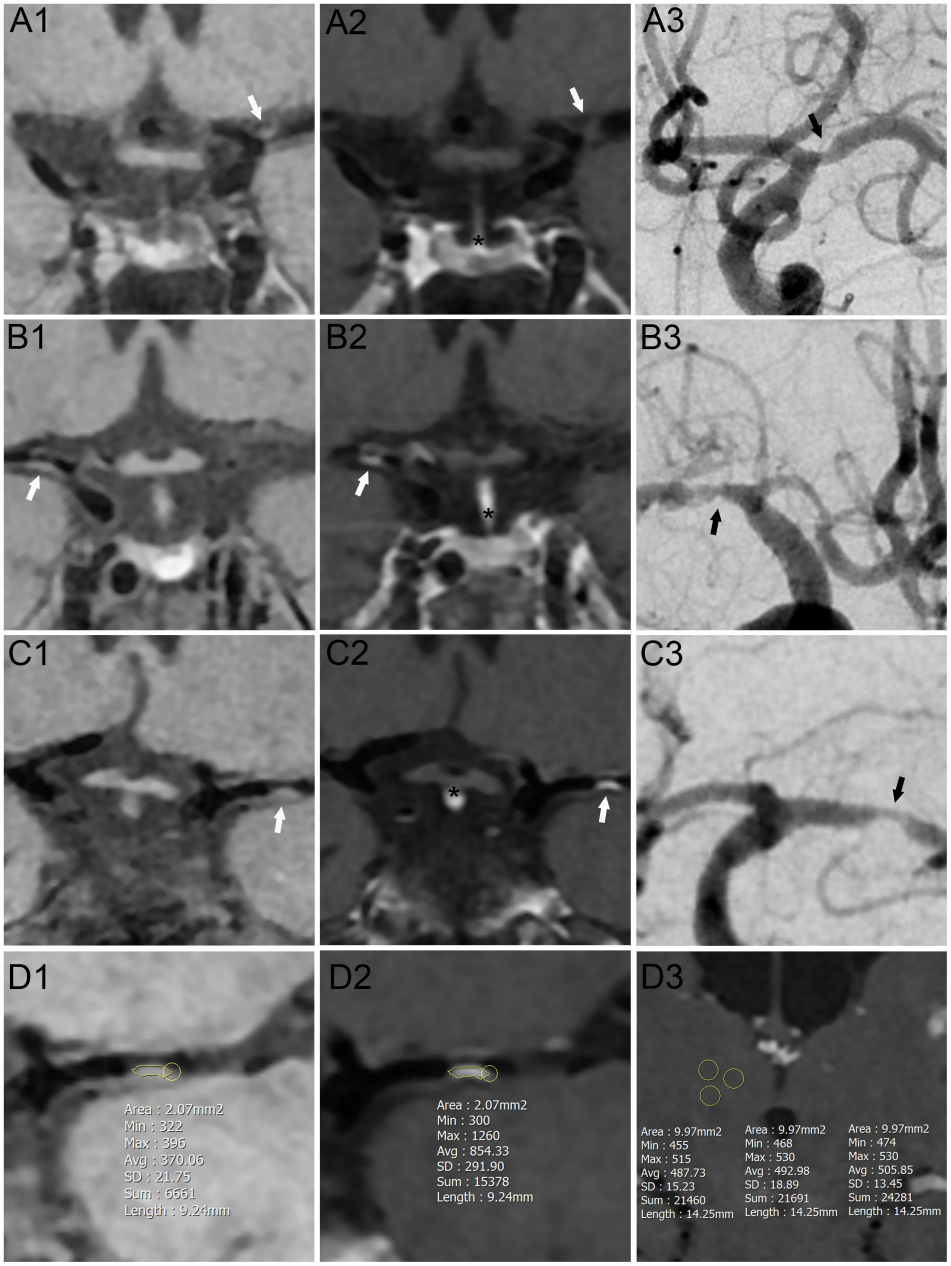
Schematic diagram of plaque enhanced assessment on contrast-enhanced high-resolution magnetic resonance imaging. **(A–C)** Qualitative method to assess the plaque enhancement. **(A)** No enhancement group. **(B)** Mild enhancement group. **(C)** Obvious enhancement group. **(D)** Quantitative method to assess the plaque enhancement. **(A1–D1)** Pre-Contrast T1-weighted sequences. **(A2–D2, D3)** Post-Contrast T1-weighted sequences. **(A3–C3)** Digital subtraction angiography sequences. White arrows: culprit plaque on contrast-enhanced high-resolution magnetic resonance imaging. Black arrows: culprit plaque on digital subtraction angiography sequences. Yellow zone: regions of interest. *: thalamic.

The clinical imaging data of the patients were analyzed independently by two experienced, blinded neurologists. Image quality was graded from 1 (poor) to 3 (excellent). Only images with a grade ≥ 2 were evaluated. We used both qualitative and quantitative methods to evaluate the degree of plaque enhancement on pre-and post-contrast T1-weighted images. First, we used a qualitative method to assess the plaque enhancement according to previously reported grading criteria (18). Signal intensity (SI) on CE-HR-MRI was compared between the culprit plaque and pituitary stalk, and the degree of enhancement was classified as follows: grade 0 = no enhancement (similar to or less than that of intracranial arterial walls without plaque in the same individual); grade 1 = mild enhancement (less than that of the pituitary stalk); and grade 2 = obvious enhancement (similar to or greater than that of the pituitary stalk) (Figures 1A–C). In cases of disagreement, the two readers reassessed the results until a consensus was reached. Culprit plaque was defined as the only lesion or the narrowest lesion within the vascular territory affected by the ischemic stroke. We then used a quantitative methodology to measure the degree of plaque enhancement (SI) according to the literature (19, 20). T1 images obtained before and after contrast administration were analyzed after being zoomed to 400% using a Picture Archiving and Communication System. A manual region of interest was linear and was placed to cover the whole area of the culprit plaque which was consistent with the stenotic segment of the intracranial artery (Figures 1D1,D2). Regions 8–10 mm^2^ in ovoid size were manually drawn around the thalamic (Figure 1D3). The measurements were repeated three times and averaged. The formula used to calculate the plaque enhancement index, i.e., the contrast ratio (CR), was as follows: CR = (post-CE T1 SI - pre-CE T1 SI) /post-CE thalamic SI. Here, CE denotes contrast enhancement, T1 denotes T1-weighted image, and SI denotes signal intensity. The degree of stenosis was determined according to the WASID criteria for digital subtraction angiography (21), which are also applied to HR-MRI, as follows: stenosis ratio = (1-narrow lumen diameter/reference lumen diameter) × 100%. The reference lumen was defined as the adjacent segment with normal appearance proximal to the stenosis.

### 2.5. Statistical analysis

Continuous variables are presented as mean and standard deviation values, or as median with interquartile range, according to the normality of the data distribution. Categorical variables are summarized as frequency (proportion). Student’s t-test was used for comparison of normally distributed variables, while the Mann–Whitney U test was used for analyzing non-normally distributed variables, and the Chi-square test or Fisher’s exact test for categorical variables. Factors that were significant (*p* < 0.05) in the univariate analysis were included in the multivariate analysis using the entry method, to examine the associations among demographic information, vascular risk factors, and laboratory test results in different plaque groups. Receiver operating characteristic (ROC) curve analysis was used to assess the predictive factors and area under the ROC curve (AUC) values were calculated. Interobserver agreement regarding the degree of plaque enhancement was assessed using a weighted kappa test. A kappa value of 0.6–0.8 was considered to indicate “substantial agreement,” and values >0.8 were considered to indicate “almost perfect agreement” (22).

All statistical analyses were carried out using SPSS (ver. 26.0; IBM Corp., Armonk, NY, United States), GraphPad Prism (ver. 8.0; GraphPad Software Inc., San Diego, CA, United States) and MedCalc software (ver. 20.1; MedCalc Software, Mariakerke, Belgium). Two-sided *p*-values ≤0.05 were considered significant.

## 3. Results

### 3.1. Baseline characteristics

**Figure 2:**
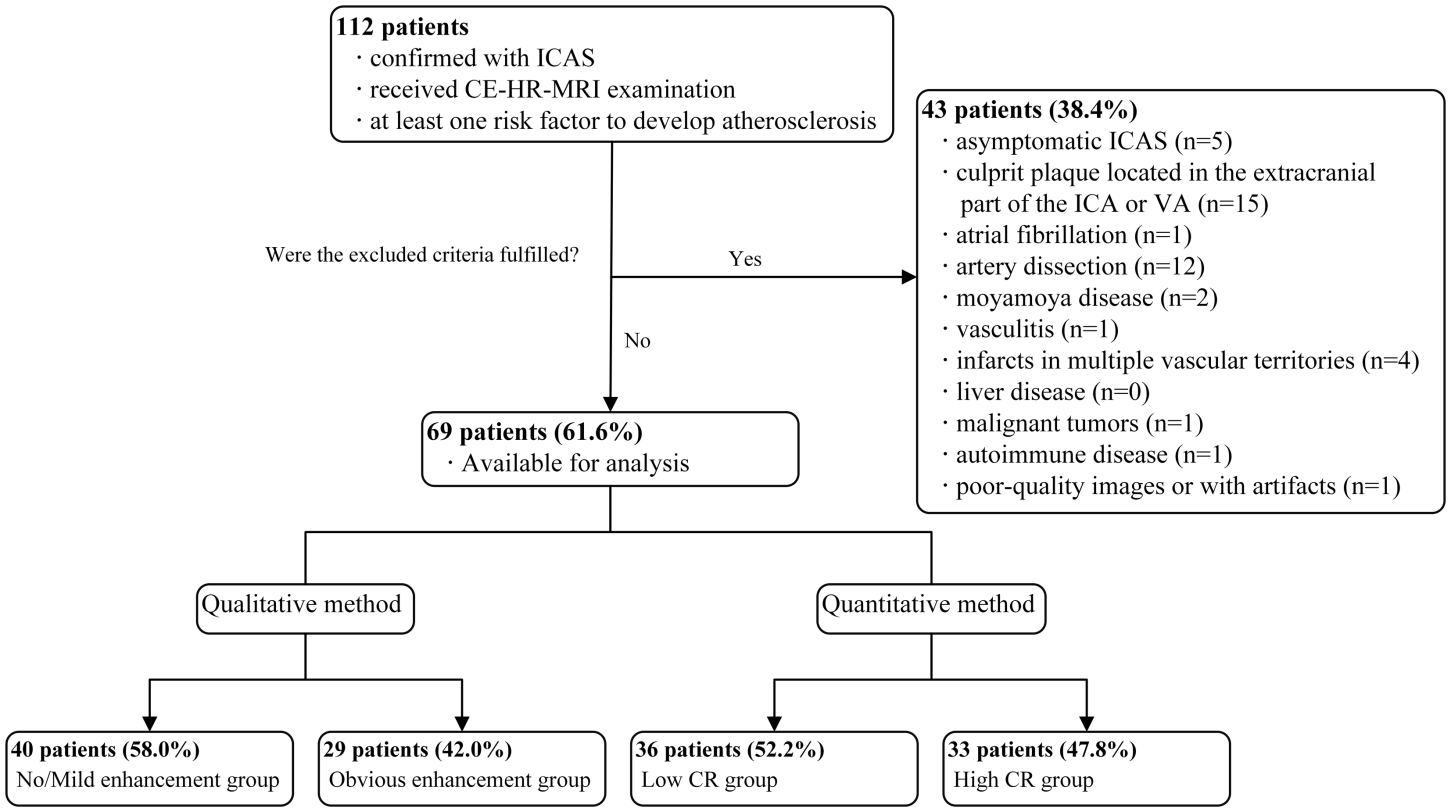
Flowchart of patient enrolment.

In total, 112 consecutive patients with ICAS who underwent CE-HR-MRI examination in the Second Affiliated Hospital of Xi’an Jiaotong University between January 2022 and September 2022 were enrolled in this study. After excluding 43 patients according to the predefined exclusion criteria, 69 patients were included in the final analysis. The study flowchart is shown in Figure 2.

The baseline characteristics of the patients are shown in Table 1. The mean age was 58.3 ± 11.6 years and there were 51 males (73.9%). Patients were classified into two groups according to the qualitative assessment of plaque enhancement: a no (*n* = 3)/mild (*n* = 37) enhancement group and an obvious enhancement group (*n* = 29). There was substantial agreement between the two observers according to the weighted kappa value (κ = 0.869, 95% confidence interval [CI]: 0.759–0.979). The groups were similar in terms of demographic data, vascular risk factors, and the degree and site of stenosis.

**Table 1:** Baseline characteristics of the plaque enhancement groups.

	Total (*n* = 69)	No/Mild enhancement group (*n* = 40)	Obvious enhancement group (*n* = 29)	*p* value
Demographic data				
Age	58.3 (11.6)	58.1 (12.4)	58.6 (10.7)	0.865
Male	51 (73.9)	29 (72.5)	22 (75.9)	0.754
History				
Smoking	36 (52.2)	20 (50.0)	16 (55.2)	0.671
Drinking	23 (33.3)	15 (37.5)	8 (27.6)	0.389
Hypertension	48 (69.6)	29 (72.5)	19 (65.5)	0.534
Diabetes mellitus	26 (37.7)	16 (40.0)	10 (34.5)	0.641
Dyslipidemia	26 (37.7)	13 (32.5)	13 (44.8)	0.297
Coronary artery disease	11 (15.9)	6 (15.0)	5 (17.2)	1.000
Interval from onset to admission	5.03 (3.00–16.12)	9.95 (3.00–19.83)	4.01 (3.00–7.91)	0.093
Degree of stenosis	85 (70–90)	80 (64–90)	90 (71–100)	0.051
Stenosis site				0.935
Anterior circulation	52 (75.4)	30 (75.0)	22 (75.9)	
Posterior circulation	17 (24.6)	10 (25.0)	7 (24.1)	

### 3.2. Laboratory tests

**Figure 3:**
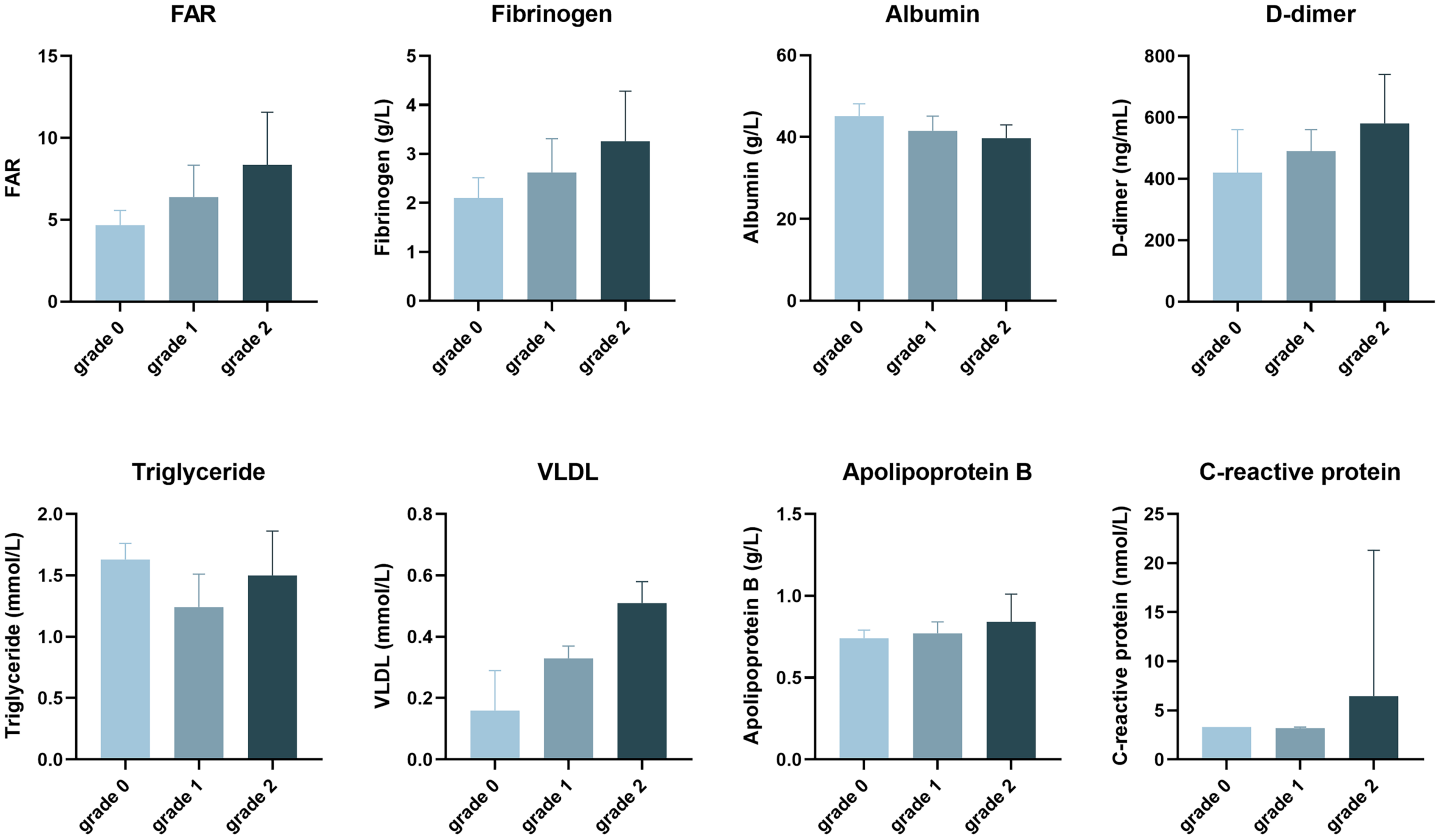
The degree of plaque enhancement of intracranial atherosclerotic stenosis patients, as revealed by laboratory tests.

The laboratory tests are shown in Table 2 and Figure 3. The triglyceride (1.50 vs. 1.26, *p* = 0.048), very low-density lipoprotein (VLDL) (0.51 vs. 0.31, *p* = 0.004), C-reactive protein (CRP, 6.44 vs. 3.25, *p* = 0.023), D-dimer (580 vs. 490, *p* = 0.013), fibrinogen (3.26 vs. 2.58, *p* = 0.002), and FAR (7.36 vs. 6.05, *p* = 0.001) were significantly higher in the obvious enhancement than no/mild enhancement group, while albumin (39.74 vs. 41.80, *p* = 0.018) was lower in the obvious enhancement than no/mild enhancement group. The other markers were not significantly different between the groups.

**Table 2:** Laboratory test results of the plaque enhancement groups.

	Total (*n* = 69)	No/Mild enhancement group (*n* = 40)	Obvious enhancement group (*n* = 29)	*p* value
Leukocyte (10^9^/L)	6.40 (5.22–8.10)	5.60 (5.02–8.04)	7.01 (5.81–8.13)	0.081
Neutrophil (10^9^/L)	3.87 (3.13–5.22)	3.81 (2.80–5.22)	4.19 (3.37–5.29)	0.458
Lymphocyte (10^9^/L)	1.67 (1.35–2.06)	1.63 (1.22–2.02)	1.70 (1.48–2.28)	0.141
Monocyte (10^9^/L)	0.43 (0.32–0.56)	0.37 (0.30–0.53)	0.44 (0.38–0.58)	0.118
Platelet (10^9^/L)	212 (175.5–256.0)	204 (167.8–256.5)	222 (191.5–263.5)	0.157
Cholesterol (mmol/L)	3.49 (3.13–4.57)	3.43 (3.13–4.22)	3.89 (3.05–4.78)	0.408
Triglyceride (mmol/L)	1.40 (1.04–1.78)	1.26 (0.97–1.63)	1.50 (1.26–1.89)	0.048*
HDL (mmol/L)	1.02 (0.21)	1.05 (0.20)	0.98 (0.23)	0.191
LDL (mmol/L)	2.17 (1.90–2.90)	2.07 (1.90–2.54)	2.37 (1.93–3.08)	0.204
VLDL (mmol/L)	0.36 (0.18–0.53)	0.31 (0.14–0.40)	0.51 (0.30–0.60)	0.004*
Apolipoprotein A1 (g/L)	1.15 (1.05–1.29)	1.15 (1.07–1.31)	1.15 (0.91–1.23)	0.334
Apolipoprotein B (g/L)	0.82 (0.25)	0.77 (0.23)	0.89 (0.26)	0.051
CRP^a^ (nmol/L)	3.30 (2.12–5.74)	3.25 (0.54–3.30)	6.44 (3.19–20.78)	0.023*
ESR^b^ (mm/H)	9 (3.0–14.0)	8 (4.0–11.3)	12 (2.0–25.5)	0.511
D-dimer (ng/mL)	530 (420–660)	490 (390.0–567.5)	580 (455.0–770.0)	0.013*
Fibrinogen (g/L)	2.88 (0.90)	2.58 (0.68)	3.26 (1.02)	0.002*
Albumin (g/L)	40.9 (3.60)	41.80 (3.65)	39.74 (3.22)	0.018*
NLR	2.42 (1.90–3.54)	2.44 (1.94–3.67)	2.36 (1.78–3.48)	0.808
LMR	4.03 (2.99–5.34)	3.95 (2.67–5.30)	4.03 (3.28–5.60)	0.903
SII	526.74 (293.6–761.50)	522.06 (338.40–723.23)	528.00 (288.46–875.58)	0.923
FAR	6.50 (5.50–8.19)	6.05 (5.15–6.88)	7.36 (6.33–10.21)	0.001*

HDL, high density lipoprotein; LDL, low density lipoprotein; VLDL, very low-density lipoprotein; CRP, C-reactive protein; ESR, Erythrocyte Sedimentation rate; NLR, neutrophil-to-lymphocyte ratio; LMR, lymphocyte-to-monocyte ratio; SII, systemic immune-inflammation index; FAR, fibrinogen-to-albumin ratio. *p–value < 0.05.

a Missing data on C-reactive protein in 29 patients (42%).

b Missing data on Erythrocyte Sedimentation rate in 30 patients (43%).

### 3.3. Multivariate regression analysis

The results of the multivariable logistic regression analysis are shown in Table 3. Factors with a value of *p* <0.05 in the univariate analysis (see Tables 1, 2) were entered into multivariable models using the entry method. After adjusting for potential confounders, the FAR and fibrinogen level were still significantly independently associated with obvious plaque enhancement (odds ratio [OR]: 1.399, 95% CI: 1.080–1.813; *p* = 0.011; and OR: 2.554, 95% CI: 1.238–5.270, *p* = 0.011, respectively).

**Table 3:** Multivariable logistic regression analysis of risk factors for obvious plaque enhancement.

Model	Adjusted OR 95%CI	*p* value
Model 1 (with FAR)		
Triglyceride (mmol/L)	0.795 (0.365–1.734)	0.795
VLDL (mmol/L)	23.333 (0.961–566.269)	0.053
D-dimer (ng/mL)	1.000 (0.999–1.001)	0.975
FAR	1.399 (1.080–1.813)	0.011*
Model 2 (with Fibrinogen)		
Triglyceride (mmol/L)	0.807 (0.373–1.747)	0.586
VLDL (mmol/L)	24.456 (1.018–587.752)	0.049*
D-dimer (ng/mL)	1.000 (0.999–1.001)	0.880
Fibrinogen (g/L)	2.554 (1.238–5.270)	0.011*
Model 3 (with Albumin)		
Triglyceride (mmol/L)	0.775 (0.349–1.723)	0.532
VLDL (mmol/L)	22.893 (1.033–507.190)	0.048*
D-dimer (ng/mL)	1.000 (0.999–1.001)	0.947
Albumin (g/L)	0.867 (0.739–1.017)	0.080

OR, odds ratio; CI, confidence interval; VLDL, very low-density lipoprotein; FAR, fibrinogen-to-albumin ratio. *p-value < 0.05.

### 3.4. Receiver operating characteristic curve analysis

**Figure 4:**
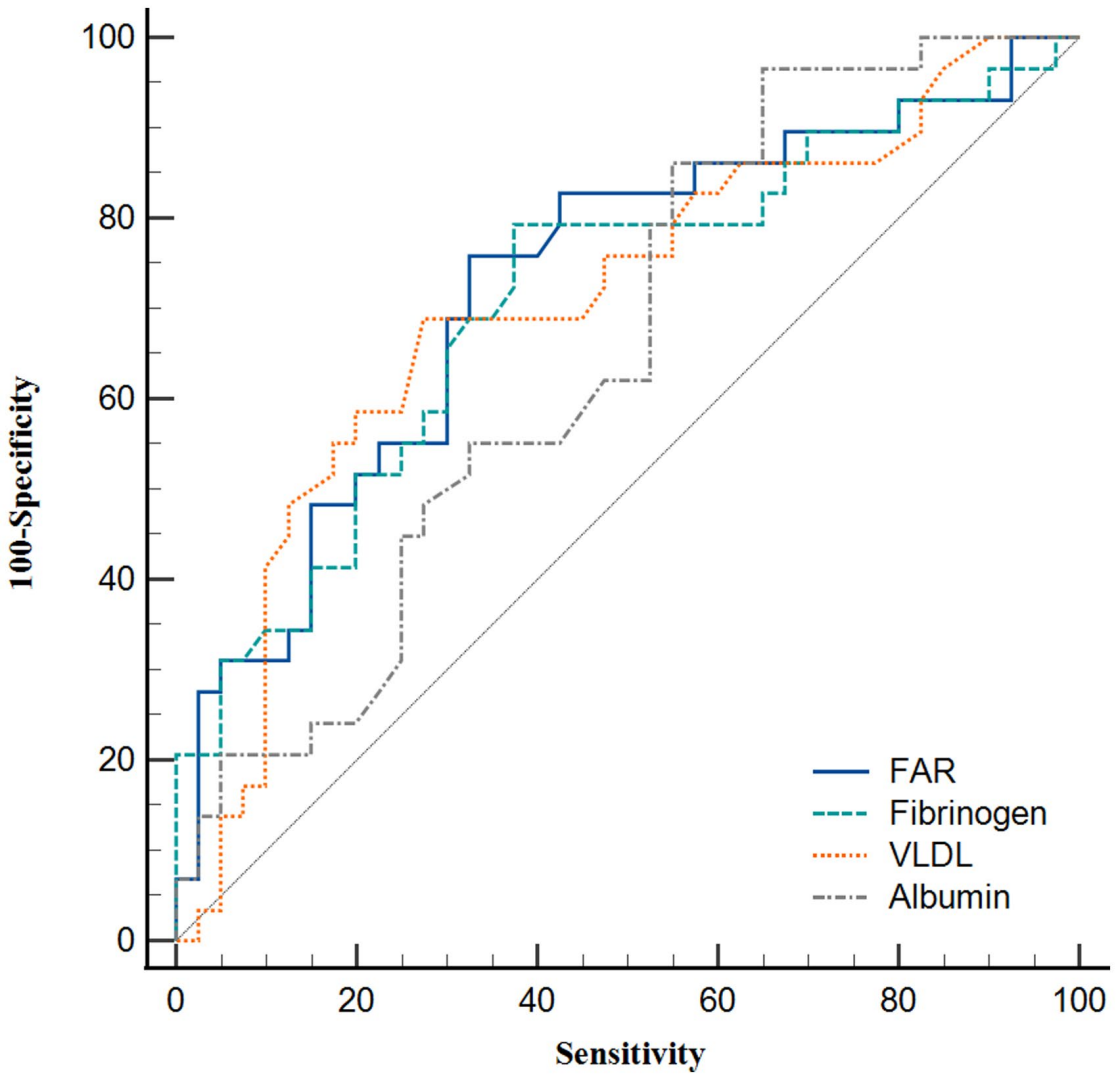
Receiver operating characteristic curve analysis showing the predictive ability of various parameters for obvious plaque enhancement.

ROC curve analysis was performed to assess the clinical sensitivity and specificity of different parameters for predicting the degree of plaque enhancement (Figure 4 and Table 4). FAR was the best biomarker for predicting obvious plaque enhancement, with an AUC of 0.726, followed by fibrinogen and VLDL (AUC = 0.711 and 0.707, respectively). The optimal cut-off for the FAR (Youden index = 6.37) had a sensitivity of 75.86% and specificity of 67.50%.

**Table 4:** Predictive value of various parameters for obvious plaque enhancement: results of receiver operating characteristic curve analysis.

	AUC (95% CI)	Cutoff	Sensitivity	Specificity	*p* value
FAR	0.726 (0.606–0.827)	6.37	75.86	67.50	<0.001*
Fibrinogen	0.711 (0.589–0.814)	2.57	79.31	62.50	0.001*
VLDL	0.707 (0.585–0.810)	0.37	68.97	72.50	0.002*
Albumin	0.653 (0.529–0.764)	44.1	96.55	35.00	0.020*

ROC, receiver operating characteristic; AUC, the area under the ROC curve; CI, confidence interval; FAR, fibrinogen-to-albumin ratio; VLDL, very low-density lipoprotein *p-value < 0.05.

We also used a quantitative method to explore the relationship between the degree of plaque enhancement and FAR (Supplementary Table S1). Patients were classified into two groups according to the median plaque CR: a low CR group (*n* = 36) and high CR group (*n* = 33). The high CR group had a significantly higher leukocyte count (7.01 vs. 5.49, *p* = 0.050), monocyte count (0.46 vs. 0.37, *p* = 0.049), fibrinogen level (3.04 vs. 2.49, *p* = 0.003), and FAR (6.99 vs. 5.94, *p* = 0.002), and a lower albumin level (39.86 vs. 41.91, *p* = 0.017). The multivariate logistic regression analysis also showed that the FAR, fibrinogen, and albumin were independently associated with plaque enhancement (Supplementary Table S2).

## 4. Discussion

This study was the first designed specifically to examine the relationship between FAR and plaque enhancement among ICAS patients. The results indicated that elevated FAR was independently associated with plaque enhancement on CE-HR-MRI, even after adjusting for all potential confounders in both qualitative and quantitative analyses.

Several studies found that intracranial plaque enhancement on CE-HR-MRI was closely related to ischemic stroke (23–25); this suggests that vulnerable and non-vulnerable plaque can be distinguished by CE-HR-MRI (8). There are several plausible reasons for atherosclerotic plaque enhancement, and the role of inflammation has attracted considerable attention for a long time. Earlier studies focused more on extracranial atherosclerotic plaque enhancement through pathohistological analysis of plaque tissue, which can easily be performed by endarterectomy. In those studies, the degree of carotid plaque enhancement was strongly associated with loose matrix content, extensive neovascularization, and inflammatory cell infiltration (10, 26), as well as an increase in serum markers of inflammation such as interleukin-6, CRP, and intercellular adhesion molecule-1 (9). However, considering the histological differences between the intracranial and extracranial arteries, the results of these studies cannot be directly applied to ICAS patients. Also, some shortcomings of CE-HR-MRI such as appointment in advance, high cost, long exam duration, injection of contrast agent, and no metal in the body, limit its use for plaque enhancement. In comparison, hematological indicators are cheap and accessible. Therefore, it is necessary to investigate the role of inflammatory markers in plaque enhancement in ICAS patients.

In recent years, many studies based on the FAR have been published. Ruan et al. (15) and Lin et al. (14) revealed that a higher FAR was an independent risk factor for hemorrhagic transformation and stroke-associated pneumonia after acute ischemic stroke. Two recent prospective observational cohort studies also demonstrated that increased FAR values were associated with a poor prognosis in patients undergoing percutaneous coronary intervention and those with ST-elevation myocardial infarction (16, 17). As described above, previous studies have shown that the FAR has superior specificity and sensitivity to fibrinogen or albumin alone. In this study, we found that the FAR was independently associated with plaque enhancement among ICAS patients. In addition, the FAR had a larger AUC value (0.726) compared with fibrinogen (0.711) and albumin alone (0.653) (Figure 4).

There are several plausible explanations for the above results. First, fibrinogen may upregulate the proinflammatory cytokines interleukin-6 and tumor necrosis factor-α, thus exacerbating vascular inflammation and endothelial dysfunction; in turn, this could result in the formation of atherosclerotic plaques and plaque vulnerability (27). Higher fibrinogen levels can also activate matrix metalloproteinase-1, which causes vascular remodeling and tissue damage (28). Second, serum albumin exerts a protective, anti-inflammatory effect by inhibiting the expression of vascular cell adhesion molecule-1 and potentiating the effects of scavenging oxygen free radicals (29). Serum albumin tends to decrease under inflammatory conditions, which cause vasodilatation and increase capillary permeability; in turn, this weakens anti-inflammatory effects and exacerbates vascular endothelial injury (30). Third, endothelial dysfunction in intraplaque microvessels within atherosclerotic lesions causes vascular leakage and an accumulation of contrast medium, and thus plaque enhancement on CE-HR-MRI (9–11). Finally, inflammation may appear prior to atherosclerotic plaque formation, even in cases without risk factors for atherosclerosis (9). Against this background, it is important to investigate the roles of serological markers of inflammation.

Two recent studies addressed certain aspects of plaque enhancement factors. The first of these studies reported that plaque enhancement was closely correlated with dyslipidemia in ICAS patients (31). This is consistent with our finding, i.e., that VLDL was independently correlated with plaque enhancement in multivariable logistic regression models 2 and 3 (see Table 3). There were slight but non-significant increases in the other serum lipid markers, such as cholesterol, triglyceride, low-density lipoprotein, and apolipoprotein B, in the obvious plaque enhancement group (see Tables 2, 3). Another retrospective study reported that a low LMR was an independent predictor of ICAS plaque enhancement (32). However, this was not the case in our study, which may be explained by the use of different inclusion, exclusion and grouping criteria.

Despite the novel findings of the present study, it also had several limitations. First, CRP, the erythrocyte sedimentation rate, and interleukin are not routinely examined in our hospital, and the missing data rate of CRP was about 40%. Thus, CRP was not included in the multivariate analysis. Interestingly, two recent papers found no correlation between CRP and plaque enhancement in ICAS patients (31, 32), in contrast to a previous study on extracranial arteries (9). Further investigation is thus needed. Second, besides the enhancement, vulnerable plaque was also associated with lipid core, fibrous cap, intraplaque hemorrhage, and calcification. The diagnosis of these characteristics requires a combination of T1WI, T2WI, TOF, PDWI, and CE-T1WI sequences. It is a shame that our hospital CE-HR-MRI lacks the T2WI sequence. Thus, these characteristics were not assessed in this study. Third, most of our inpatients had symptomatic ICAS (ischemic stroke or TIA), and the proportion of such cases in the no enhancement plaque group was very low. Hence, we had to combine the no and mild enhancement groups, which may have limited the predictive ability of the FAR.

## 5. Conclusion

In the present study, the FAR value was an independent predictor of the degree of plaque enhancement in patients with ICAS. The FAR has potential as a serological biomarker of intracranial atherosclerotic plaque vulnerability.

## Data availability statement

The raw data supporting the conclusions of this article will be made available by the authors, without undue reservation.

## Ethics statement

The studies involving human participants were reviewed and approved by the Ethics Committee of the Second Affiliated Hospital of Xi’an Jiaotong University. Written informed consent for participation was not required for this study in accordance with the national legislation and the institutional requirements.

## Author contributions

YL: conceptualization, writing – original draft preparation, and writing – review & editing. YF and GZ: conceptualization and writing – review & editing. RL and MD: data curation and writing – review & editing. TL and LiZ: formal analysis and writing – review & editing. JL and ZL: investigation and writing – review & editing. YY and XW: visualization and writing – review & editing. YJ, HW, WH, and LeZ: writing – review & editing. All authors contributed to the article and approved the submitted version.

## Funding

This research was supported by the Shaanxi Provincial Key Research and Development Project (No. 2019ZDLSF01-04) and the National Natural Science Foundation (No. 81971116), China.

## Conflict of interest

The authors declare that the research was conducted in the absence of any commercial or financial relationships that could be construed as a potential conflict of interest.

## Publisher’s note

All claims expressed in this article are solely those of the authors and do not necessarily represent those of their affiliated organizations, or those of the publisher, the editors and the reviewers. Any product that may be evaluated in this article, or claim that may be made by its manufacturer, is not guaranteed or endorsed by the publisher.
