# Supplementary material for: The fibrinogen-to-albumin ratio is associated with intracranial atherosclerosis plaque enhancement on contrast-enhanced high-resolution magnetic resonance imaging
Source: Front Neurol. 2023 May 24;14:1153171. doi: 10.3389/fneur.2023.1153171 (PMC10249607; doi:10.3389/fneur.2023.1153171)
Supplement: Supplementary file 1 [file Table_1.docx]

Supplementary Material

The fibrinogen-to-albumin ratio is associated with intracranial atherosclerosis plaque enhancement on contrast-enhanced high-resolution magnetic resonance imaging

**Ye Li^1†^, Yuxuan Feng^1†^, Rui Liu^2^, Meijuan Dang^1^, Tao Li^1^, Lili Zhao^1^, Jialiang Lu^1^, Ziwei Lu^1^, Yang Yang^1^, Xiaoya Wang^1^, Yating Jian^1^, Heying Wang^1^, Wei Huang^3^, Lei Zhang^1^, Guilian Zhang^1*^**

^1^Department of Neurology, the Second Affiliated Hospital of Xi'an Jiaotong University, Xi'an, Shaanxi, China

^2^Department of Neurology, the First Hospital of Yulin, Yulin, Shaanxi, China

^3^Department of Medical Imaging, the Second Affiliated Hospital of Xi'an Jiaotong University, Xi'an, Shaanxi, China

**^†^These authors contributed equally to this work and share first authorship**

*** Correspondence:**Guilian Zhang
zhgl_2006@xjtu.edu.cn

**Table S1** Patients’ baseline characteristics and laboratory test according to the median of plaque CR

|  | Total  (n=69) | Low CR group  (n=36) | High CR group  (n=33) | *P* value |
| --- | --- | --- | --- | --- |
| Demographic data |  |  |  |  |
| Age | 58.3 (11.6) | 57.6 (12.8) | 59.1 (10.3) | 0.608 |
| Male | 51 (73.9) | 26 (72.2) | 25 (75.8) | 0.738 |
| History |  |  |  |  |
| Smoking | 36 (52.2) | 18 (50.0) | 18 (54.5) | 0.706 |
| Drinking | 23 (33.3) | 13 (36.1) | 10 (30.3) | 0.609 |
| Hypertension | 48 (69.6) | 24 (66.7) | 24 (72.7) | 0.585 |
| Diabetes mellitus | 26 (37.7) | 15 (41.7) | 11 (33.3) | 0.475 |
| Dyslipidemia | 26 (37.7) | 13 (36.1) | 13 (39.4) | 0.779 |
| Coronary artery disease | 11 (15.9) | 7 (19.4) | 4 (12.1) | 0.406 |
| Degree of stenosis | 85 (70‒90) | 82 (62‒90) | 85 (71‒90) | 0.226 |
| Stenosis site |  |  |  | 0.233 |
| Anterior circulation | 52 (75.4) | 25 (69.4) | 27 (81.8) |  |
| Posterior circulation | 17 (24.6) | 11 (30.6) | 6 (18.2) |  |
| Laboratory test |  |  |  |  |
| Leukocyte (10^9^ /L) | 6.40 (5.22‒8.10) | 5.49 (5.02‒8.03) | 7.01 (5.74‒8.21) | 0.050* |
| Neutrophil (10^9^/L) | 3.87 (3.13‒5.22) | 3.66 (2.76‒5.11) | 4.20 (3.49‒5.48) | 0.104 |
| Lymphocyte (10^9^/L) | 1.67 (1.35‒2.06) | 1.65 (1.27‒2.02) | 1.67 (1.43‒2.09) | 0.585 |
| Monocyte (10^9^/L) | 0.43 (0.32‒0.56) | 0.37 (0.30‒0.49) | 0.46 (0.36‒0.58) | 0.049* |
| Platelet (10^9^ /L) | 212.0 (175.5‒256.0) | 199.5 (164.8‒254.3) | 222.0 (191.5‒ 276.0) | 0.078 |
| Cholesterol (mmol/L) | 3.49 (3.13‒4.57) | 3.45 (3.13‒4.34) | 3.52 (3.12‒4.74) | 0.532 |
| Triglyceride (mmol/L) | 1.40 (1.04‒1.78) | 1.38 (0.98‒1.70) | 1.41 (1.07‒1.84) | 0.397 |
| HDL (mmol/L) | 1.02 (0.21) | 1.03 (0.18) | 1.01 (0.24) | 0.650 |
| LDL (mmol/L) | 2.17 (1.90‒2.90) | 2.10 (1.90‒2.77) | 2.29 (1.90‒3.05) | 0.532 |
| VLDL (mmol/L) | 0.36 (0.18‒0.53) | 0.35 (0.14‒0.48) | 0.38 (0.22‒0.58) | 0.163 |
| Apolipoprotein A1 (g/L) | 1.15 (1.05‒1.29) | 1.14 (1.07‒1.29) | 1.15 (0.97‒1.26) | 0.674 |
| Apolipoprotein B (g/L) | 0.82 (0.25) | 0.80 (0.24) | 0.84 (0.25) | 0.427 |
| CRP^a^ (nmol/L) | 3.30 (2.12‒5.74) | 3.30 (3.19‒4.00) | 3.30 (0.81‒13.49) | 0.657 |
| ESR^b^ (mm/H) | 9 (3.0‒14.0) | 8 (4.0‒13.0) | 9.0 (2.3‒23.8) | 0.699 |
| D‒dimer (ng/mL) | 530 (420‒660) | 490 (395‒578) | 560 (435‒770) | 0.072 |
| Fibrinogen (g/L) | 2.69 (2.34‒3.2) | 2.49 (2.19‒2.87) | 3.04 (2.58‒3.66) | 0.003* |
| Albumin (g/L) | 40.93 (3.60) | 41.91 (3.75) | 39.86 (3.14) | 0.017* |
| NLR | 2.42 (1.90‒3.54) | 2.27 (1.72‒3.40) | 2.67 (2.01‒3.67) | 0.216 |
| LMR | 4.03 (2.99‒5.34) | 4.36 (3.07‒5.30) | 3.66 (2.93‒5.60) | 0.442 |
| SII | 526.74 (293.6‒761.50) | 453.40 (283.41‒688.83) | 570.33 (361.94‒833.51) | 0.133 |
| FAR | 6.50 (5.50‒8.19) | 5.94 (5.15‒6.88) | 6.99 (6.22‒9.63) | 0.002* |

Abbreviations: CR, contrast ratio; HDL, high density lipoprotein; LDL, low density lipoprotein; VLDL, very low-density lipoprotein; CRP, C-reactive protein; ESR, Erythrocyte Sedimentation rate; NLR, neutrophil-to-lymphocyte ratio; LMR, lymphocyte-to-monocyte ratio; SII, systemic immune-inflammation index; FAR, fibrinogen-to-albumin ratio.

* P‒value <0.05

^a^ Missing data on CRP in 29 patients (42%)

^b^ Missing data on ESR in 30 patients (43%)

**Table S2** Multivariable logistic regression analysis for risk factors of High plaque CR

| Model | Adjusted OR 95%CI | *P* value |
| --- | --- | --- |
| Model 1 (with FAR) |  |  |
| Monocyte (10^9^/L) | 0.838 (0.031‒22.610) | 0.916 |
| Leukocyte (10^9^ /L) | 1.203 (0.852‒1.697) | 0.294 |
| FAR | 1.373 (1.063‒1.772) | 0.015* |
| Model 2 (with Fibrinogen) |  |  |
| Monocyte (10^9^/L) | 1.065 (0.040‒28.109) | 0.970 |
| Leukocyte (10^9^ /L) | 1.180 (0.835‒1.667) | 0.349 |
| Fibrinogen (g/L) | 2.318 (1.144‒4.695) | 0.020* |
| Model 3 (with Albumin) |  |  |
| Monocyte (10^9^/L) | 0.729 (0.028‒19.055) | 0.849 |
| Leukocyte (10^9^ /L) | 1.270 (0.917‒1.758) | 0.151 |
| Albumin (g/L) | 0.845 (0.723‒0.987) | 0.034* |

Abbreviation: CR, contrast ratio; OR, odds ratio; CI, confidence interval; FAR, fibrinogen-to-albumin ratio

*P‒value <0.05
